# Monolithic Integrated OLED–OPD Unit for Point-of-Need Nitrite Sensing

**DOI:** 10.3390/s22030910

**Published:** 2022-01-25

**Authors:** Igor Titov, Markus Köpke, Martina Gerken

**Affiliations:** Integrated Systems and Photonics, Faculty of Engineering, Kiel University, 24118 Kiel, Germany; mko@tf.uni-kiel.de (M.K.); mge@tf.uni-kiel.de (M.G.)

**Keywords:** sensor systems, photonic devices, nitrite measurement, Griess reagent, OLED, OPD, monolithic integration

## Abstract

In this study, we present a highly integrated design of organic optoelectronic devices for Point-of-Need (PON) nitrite (${\mathrm{NO}}_{2}{}^{-}$) measurement. The spectrophotometric investigation of nitrite concentration was performed utilizing the popular Griess reagent and a reflection-based photometric unit with an organic light emitting diode (OLED) and an organic photodetector (OPD). In this approach a nitrite concentration dependent amount of azo dye is formed, which absorbs light around ~540 nm. The organic devices are designed for sensitive detection of absorption changes caused by the presence of this azo dye without the need of a spectrometer. Using a green emitting TCTA:Ir(mppy)_3_ OLED (peaking at ~512 nm) and a DMQA:DCV3T OPD with a maximum sensitivity around 530 nm, we successfully demonstrated the operation of the OLED–OPD pair for nitrite sensing with a low limit of detection 46 µg/L (1.0 µM) and a linearity of 99%. The hybrid integration of an OLED and an OPD with 0.5 mm × 0.5 mm device sizes and a gap of 0.9 mm is a first step towards a highly compact, low cost and highly commercially viable PON analytic platform. To our knowledge, this is the first demonstration of a fully organic-semiconductor-based monolithic integrated platform for real-time PON photometric nitrite analysis.

## 1. Introduction

Nitrates and nitrites play an essential role for plant growth in agriculture. Thus, they are a major component of inorganic fertilizers [1]. Their high solubility in water results in a particularly critical exceedance of limits in the ground water [2]. The accumulation of agricultural chemicals in the groundwater is a well-known problem. In 1989, Hallberg reported about 39 pesticides in the groundwater of 34 states or provinces of the United States with nitrate as one of the common agricultural chemicals [3]. The exceedance of nitrates and nitrites limits in environmental and physiological systems have adverse effects on animal and human health. The World Health Organization (WHO) set a guideline value of 3 mg/L nitrite in water. Recent studies reported carcinogenic effects, methemoglobinemia, and detrimental effects on the thyroid gland and other organs, associated with the ingestion of high concentrations of nitrate and nitrite due to the high toxicity. Infants are particularly susceptible to methemoglobin formation due to reduction in nitrite. In contrast to hemoglobin, methemoglobin is unable to transport oxygen to the tissues. This condition causes cyanosis or asphyxia. Thus, the detection of nitrite in samples such as water, urine, saliva or blood plasma is crucial and has been reported by many different research groups [4]. 

Current methods for nitrite ion detection include electrochemical methods (including voltametric, potentiometric and impedimetric electrodes), spectrophotometry, spectrofluorimetry and ion chromatography [5,6,7,8,9]. Electrochemical sensors are popular due to the potential low cost, portability and simple fabrication. Despite the wide application areas there are still some limitations. Current research on electrochemical sensors focusses on improving the limit of detection and reducing the cross-sensitivity of the electrodes [10]. Here, we focus on spectrophotometry as the most common method for nitrite detection due to its easy procedure, low detection limits, high selectivity and low cost. In particular, the assay based on the Griess reagent for colorimetric nitrite sensing is very popular due to its high stability, selectivity and sensitivity [11]. Early work has been conducted by Petsul et al. using the Griess reaction and an external light emitting diode (LED) with a spectrometer for the absorbance measurements, achieving a limit of detection (LOD) of 0.2 mM [12]. Since then, a variety of different sensor systems with different optical device configurations have been reported. Colorimetric nitrite sensors mostly use LEDs as the light source. Devices such as CCD cameras, spectrometers, smartphone cameras, photomultiplier tubes (PMT) and photodiodes (PD) were recently successfully integrated on the detector side [13,14,15,16,17,18,19,20,21,22]. While very impressive results are achieved with the compact integration of the inorganic optoelectronic devices and microfluidic-based approaches, these systems are still rather bulky. For low-cost portable Point-of-Need (PON) solutions, further miniaturization and a simple fabrication method are needed. 

To overcome the aforementioned limitations, the integration of organic light emitting diodes (OLEDs) and organic photodetectors (OPDs) promises compact and low-cost optical detection units as hybrid integrated sensors. Recently, very impressive results have been achieved with fabrication of OLEDs and OPDs on flexible and rigid substrates. The devices have been proposed as wearable sensors for health monitoring and Point-of-Need analysis applications [23,24,25,26,27,28,29]. Due to the thermal evaporation-based device fabrication technique, all sensing elements are permanently aligned, allowing a high degree of miniaturization. These units can be easily laminated to a microfluidic system for sensing applications in a liquid [30,31,32,33,34].

Based on our experience in biomedical lab-on-chip systems for multiplexed detection [35,36] and monolithic integrated organic optoelectronic devices [37,38], we aim at developing a highly integrated nitrite sensor for Point-of-Need analysis. In this work we present, to the best of our knowledge, the first demonstration of a fully organic optoelectronic system for photometric nitrite sensing based on the Griess reagent with a reflection-type architecture. The OLED and the OPD are successfully monolithically integrated and have a device size of 0.5 mm × 0.5 mm each. The gap between the devices is 0.9 mm. Due to the monolithic integration and a simple fabrication process, the scalability of parallel fabricated sensor units is promising, leading to low cost and potentially disposable PON systems. As a consequence, the commercial viability can be significantly increased.

In the Experimental Section 2, we present in Section 2.1 the OLED–OPD device design and the fabrication procedure of the organic stacks. The experimental characterization of the OPD and OLED devices is given in Section 2.3. Section 2.4 presents the design of the nitrite sensing platform followed by the nitrite sensing experiments in Section 2.5. Conclusions are given in Section 3. 

## 2. Experimental Section

### 2.1. OLED–OPD Matrix Fabrication

**Figure 1:**
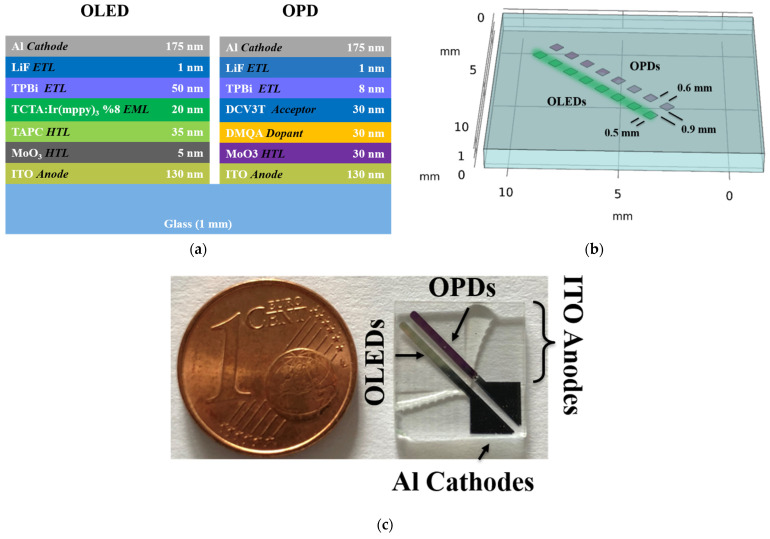
(**a**) Schematic diagram of the OLED and OPD stacks on a single glass substrate; (**b**) geometrical model of the OLED–OPD unit layout with 8 units on one test chip; (**c**) photograph of a fabricated test chip.

Figure 1a–c show the organic stacks, a geometrical schematic and a photograph of the test chip with 8 OLED–OPD detection units. The realized TCTA:Ir(mppy)_3_ OLEDs and DMQA:DCV3T OPDs were fabricated on a 25 mm × 25 mm glass substrate with a thickness of 1 mm. Each substrate contained 4 test chips, which were separated afterwards to 12.5 mm × 12.5 mm sensing units by a wafer saw. Here, each test chip had 8 OLED–OPD detection units. The green emitting OLED (peaking at ~512 nm) pixels fabrication was carried out on indium-doped tin oxide (ITO) covered glass by thermally evaporating organic materials. The ITO layer was structured via ultraviolet (UV) photolithography and wet etching to form the 130 nm thin anodes. The organic layers, in sequence, were the hole transport layer of 5 nm molybdenum trioxide (MoO_3_) and 35 nm 1,1-bis[(di-4-tolylamino)phenyl]cyclohexane (TAPC), then for the emission layer 20 nm of tris(4-carbazoyl-9-ylphenyl)amine (TCTA) doped with 8% Tris[2-(p-tolyl)pyridine]iridium(III) (Ir(mppy)_3_) to generate a highly efficient green-emitting OLED. As the electron transport layer (ETL) and hole blocking layer (HBL) a film of 50 nm 2,2′,2″-(1,3,5-Benzinetriyl)-tris(1-phenyl-1-H-benzimidazole) (TPBi) was deposited followed by the cathode pads consisting of 1 nm lithium fluoride (LiF) and 175 nm aluminum (Al). Consequently, the OLED pixels were defined by the overlapping regions of mutually perpendicular ITO and Al pads.

The OPD structure starts with a 30 nm molybdenum trioxide (MoO3) layer deposited on the indium-tin-oxide (ITO) coated glass. The organic bilayer structure is composed of 30 nm N,N-Dimethyl quinacridone (DMQA) as the common donor and 30 nm 3′,4′-Dibutyl-5,5″-bis(dicyanovinyl)-2,2′:5′,2″-terthiophene (DCV3T) as the acceptor layer. Next, 8 nm 2,2′,2″-(1,3,5-Benzinetriyl)-tris(1-phenyl-1-H-benzimidazole) (TPBi) was evaporated followed by 1 nm lithium fluoride (LiF) and 175 nm aluminum (Al) to form the cathode pads. The fabricated devices were encapsulated in a nitrogen-filled glovebox to prevent degradation of the organic layers upon measurements outside the glovebox.

### 2.2. Chemicals and Materials

Following materials were used as the organic components of the devices: 1,1-bis[(di-4-tolylamino)phenyl]cyclohexane (TAPC, CAS-No: 58473-78-2, Merck KGaA, Darmstadt, Germany), tris(4-carbazoyl-9-ylphenyl)amine (TCTA, CAS-No: 139092-78-7, Merck KGaA, Darmstadt, Germany), 2,2′,2″-(1,3,5-Benzinetriyl)-tris(1-phenyl-1-H-benzimidazole) (TPBi, CAS-No: 192198-85-9, Luminescence Technology Corp., New Taipei City, Taiwan), Tris[2-(p-tolyl)pyridine]iridium(III) (Ir(mppy)_3_, CAS-No: 149005-33-4, Luminescence Technology Corp., New Taipei City, Taiwan), N,N-Dimethyl quinacridone (DMQA, CAS-No: 19205-19-7, Lumtec), 3′,4′-Dibutyl-5,5″-bis(dicyanovinyl)-2,2′:5′,2″-terthiophene (DCV3T, CAS-No: 908588-68-1, Luminescence Technology Corp., New Taipei City, Taiwan), molybdenum trioxide (MoO_3_, CAS-No: 1313-27-5, Merck KGaA, Darmstadt, Germany), lithium fluoride (LiF, CAS-No: 7789-24-4, Merck KGaA, Darmstadt, Germany), aluminum (Al, CAS-No: 7429-90-5, Merck KGaA, Darmstadt, Germany).

Nitrite standard solutions were diluted from 200 mg/L sodium nitrite (NaNO_2_) prior to use. The stock solution was prepared by dissolving 0.3 g sodium nitrite (CAS-No: 908588-68-1, Merck KGaA, Darmstadt, Germany) in deionized water. A nitrite essay kit (MAK376, Merck KGaA, Darmstadt, Germany) was used to prepare the Griess reagent.

### 2.3. Device Characterization

**Figure 2:**
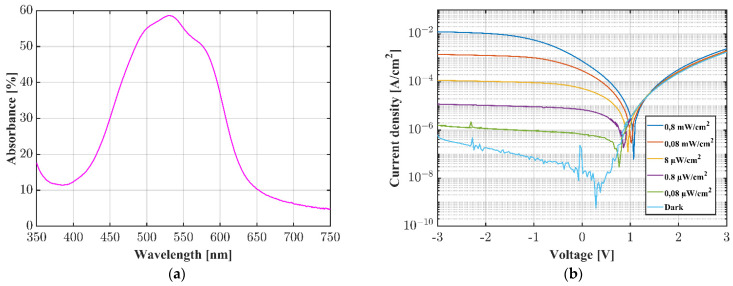
Optical and electrical characterization of the DMQA.DCV3T OPD. (**a**) UV-Vis measurement of 60 nm DMQA:DCV3T absorptive bilayer; (**b**) current density–voltage characteristics in the dark and under illumination with a subsequently filtered 520 nm laser.

The characteristics of one OPD are shown in Figure 2a,b. The absorption spectrum of the organic donor–acceptor bilayer composite was determined using a UV-Vis spectrometer (Lambda 800, PerkinElmer, Waltham, MA, USA), showing the highest sensitivity between 500 and 550 nm. The current–voltage characteristics were performed by a source measure unit (SourceMeter 2450, Keithley Instruments, Cleveland, OH, USA) and a 520 nm collimated laser module (CPS520, 4.5 mW, Thorlabs GmbH, Lübeck, Germany). The OPD shows typical diode characteristics and a good sensitivity. The measured photocurrent was 15.4 nA under dark conditions and 0.7 mA for an incident light power of ~0.8 mW/cm^2^ at the wavelength of 520 nm at 0 V.

**Figure 3:**
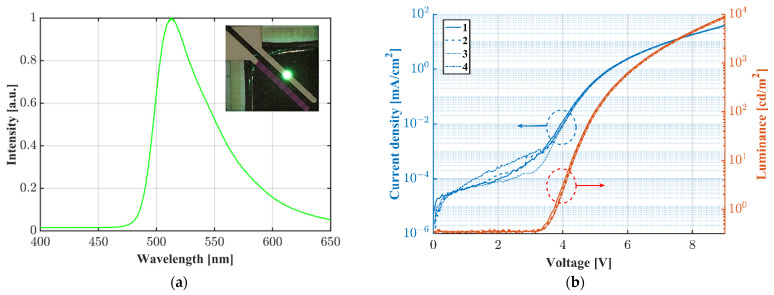
(**a**) Normalized spectrum of the green emitting (peaking at ~512 nm) Ir(mppy)_3_ OLED as an example; (**b**) current density and luminance vs. voltage characteristics of the fabricated bottom-emitting OLED.

The OLED emission spectrum was measured with a spectrometer (iHR320, Horiba, Kyoto, Japan) by using an optical fiber placed right above the OLED pixel. For optical and electrical characterization, we used a commercial calibrated Si-photodiode (FDS1010, Thorlabs GmbH, Lübeck, Germany) and two source measure units (SourceMeter 2450 and 2400, Keithley Instruments, Cleveland, OH, USA). The bottom-emitting OLED was placed on top of the large-area photodiode to collect the outcoupled light. The current density and the luminance were calculated with the recorded OLED current and the Si-photodiode photocurrent induced by the OLED. Figure 3a shows the spectrum of the green-emitting OLED. The peak wavelength is located at ~512 nm. The inset shows a photograph of an OLED pixel operated at 9 V. Figure 3b shows the electrical and optical characterization of the Ir(mppy)_3_ OLED. The onset voltage is approximately 3.3 V. The measurement was performed 4 times in sequence and showed good repeatability.

It is stressed that the spectrometer is only used for device characterization and reference measurements. No spectrometer is needed for the final nitrite-measurement system.

### 2.4. Design of the Test Chamber

**Figure 4:**
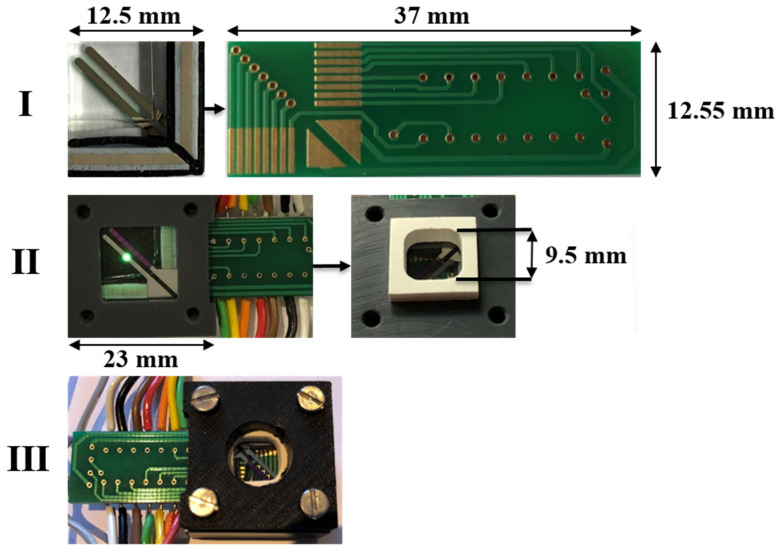
Nitrite measurement detection setup. The system is housing an OLED–OPD matrix, contacted with elastomeric ZEBRA^®^ connectors and a printed circuit board (PCB) with mirrored electrodes design (**Step I**). The organic platform and the PCB are pressed to each other with a milled mount. The fluid chamber is sealed with a rubber seal, pressed on top of the organic OLED–OPD chip (**Step II**). Finally, the setup was used with a drilled cap for adding and removing liquids with a pipette (**Step III**).

For proof-of-principle demonstration of nitrite sensing with the OLED–OPD unit, we realized a test setup for contacting and fluid application. The OLED–OPD units were contacted with a printed circuit board (PCB) and elastomeric ZEBRA^®^ connectors. The proof of concept was performed with a custom rubber seal covered with a cap to ensure tightness and avoid leakage of the chemicals. The setup was used with a drilled cap (Figure 4III) and the liquids were filled into the chamber with a pipette. The measurements were performed with two source measure units for OLED supplying and recording the photocurrent of the OPD in a darkened environment.

### 2.5. Nitrite Sensing with OLED–OPD Matrix

**Figure 5:**
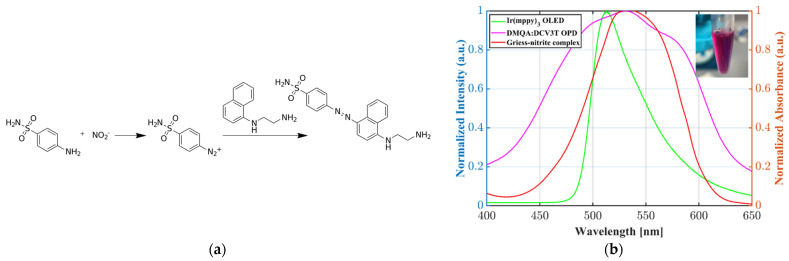
(**a**) Schematic of the Griess reaction for spectrophotometric quantification of the nitrite concentration; (**b**) overview of the OLED emission spectrum as well as the absorption spectra of the OPD and the Griess-reaction-based azo dye. The chosen organic composition of the OLED–OPD system has overlapping emission and absorbance spectra for the highest nitrite sensitivity. The inset shows, as an example, strong absorbance at around 540 nm for a nitrite standard sample of 30 mg/L.

The photometric nitrite sensing was performed with a reagent based on the Griess reaction. Figure 5a shows the chemical transformation of N-(1-naphthyl)ethylenediamine dihydrochloride (NED) and sulfanilamide in an acidic solution. Sulfanilamide reacts with the nitrite ion and forms a diazonium salt. The azo dye formation occurs due to the reaction with NED. The absorbance around 540 nm strongly depends on the initial nitrite concentration.

**Figure 6:**
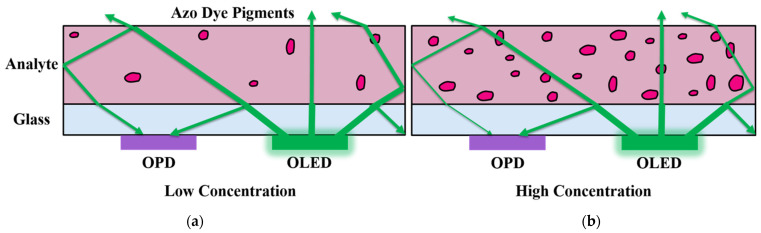
Schematic of the azo dye concentration-dependent absorbance of OLED light inside the analyte. (**a**) The light propagated partly inside the glass substrate and was partly reflected back onto the OPD; (**b**) for higher concentrations of nitrite standard sample and consequently higher concentrations of the azo dye the light showed increased absorption inside the chamber at the azo dye pigments. Thus, only a fraction of initial amount of light was reflected back onto the OPD. The photocurrent decreased as a consequence.

As depicted in Figure 5b, we chose the organic composition of the OLED–OPD matrix for the highest absorbance of the green OLED light on the OPD. Due to total internal reflection (TIR) the light propagated partly inside the glass substrate and induced photocurrent in the OPD, since the OLED is an extended device with isotropic emission. The following Figure 6 shows a schematic of the expected azo dye concentration dependent signal change of the OLED–OPD matrix unit. In Figure 6a the light propagated partly inside the glass substrate and was partly reflected back onto the OPD after traveling inside the analyte. For lower sample concentration the partly absorbed light was reflected multiple times at the surfaces inside the liquid chamber and was partly reflected back onto the OPD. For higher concentration (Figure 6b) of nitrite standard sample and, consequently, higher concentration of the azo dye, the absorbance of the light inside the chamber increased. Thus, only a fraction of initial emitted amount of light was reflected back onto the OPD and the photocurrent consequently decreased.

The nitrite sensing experiments were performed with nitrite standard solutions at room temperature prepared 10 min prior to use. Although the azo dye formation was near instantaneous, according to the datasheet the reaction was accomplished after an incubation time of 10 min. The signal was stable for 1 h after adding the reagents. Brizzolari et al. reported a reaction time of 15 min to achieve the most stable results [39]. Pai and Yang evaluated the azo forming kinetics for different concentration of NED and the final acidity [40]. The evaluated *t*(90%) values were measured to be in the region of <1 min. The OLED was operated at constant current mode at 60 µA during all measurements. The photocurrent of the OPD was recorded at zero bias.

**Figure 7:**
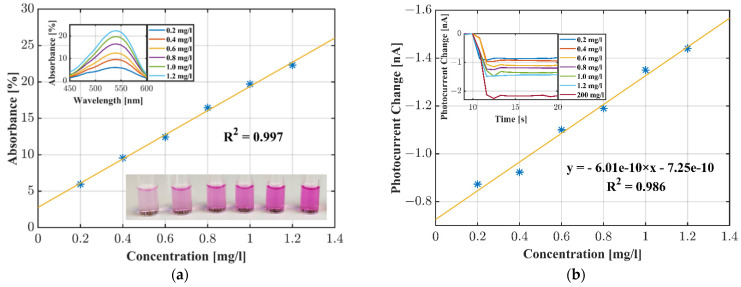
(**a**) UV-Vis reference measurement evaluated at 540 nm; (**b**) nitrite measurement and the calibration plot for the nitrite standard solutions obtained with the OLED–OPD unit and the azo dye absorbance.

Figure 7a,b shows the measurement of the premixed Griess reagent and nitrite standard solutions. Prior to the measurements with the OLED–OPD unit we tested the nitrite samples with a UV-Vis spectrometer (Lambda 800, PerkinElmer, Waltham, MA, USA). Figure 7a shows a calibration plot with a linearity of 99% as a reference. Figure 7b shows the measurement with the investigated OLED–OPD sensing unit. We started each measurement with an empty fluid chamber and subsequently added samples of 200 µL ranging from 0.2 to 1.2 mg/L nitrite with a pipette. The Griess complex recorded the signal response in real time. The signal was a superposition of waveguided light, stray light and light passing through the liquid chamber. The detected intensity depended on the absorbance of light by the azo dye. The observed decrease in the photocurrent upon addition of the nitrite solution is shown in the inset of Figure 7b. For example, the photocurrent for the empty chamber was 2.46 nA and after addition of a 1.0 mg/L nitrite solution the photo current changed to 1.10 nA. Thus, the absolute value of the current was reduced by 1.36 nA. The calibration function obtained from these measurements was found to be linear up to 1.2 mg/L, offering linearity of approximately 99%. The sensitivity was calculated as S = 0.6 nA/(mg/L). The standard deviation current noise level was determined as σ = 9.23 pA. This yielded a limit of detection (LOD) of LOD = 3 × σ/S = 46 µg/L (1.0 µM).

The system shows the highest photocurrent induced by the OLED light in the absence of the analyte in the fluidic chamber. Injecting the azo dye concentrated sample inside the chamber resulted in decreasing photocurrent due to the superposition of the refractive index change and the absorbance of the light at azo dye pigments. Since the critical angle for total internal reflection (TIR) inside the glass substrate increases for liquid analytes on the OLED–OPD matrix surface, the amount of light entering the analyte also increased and resulted in less guided stray light inside the glass substrate. For constant refractive indices of the analytes, the offset of the photocurrent was also constant and was consequently neglected during the measurement. The OPD signal at high absorption is determined with a highly concentrated nitrite sample (200 mg/L) to be 2.17 nA. As a further verification of the absorption inside the analyte, we evaluated the influence on the OPD photocurrent by adding a mirror on top of the OLED–OPD measurement unit. Due to reflection of the light back to the OPD we observed a small increase in the photocurrent for low concentrations of the nitrite samples, whereas adding a mirror for high nitrite concentrations resulted in no enhancement of the photocurrent. This is due to the high absorption of the high azo dye concentration.

Table 1 shows a comparison between our investigated OLED–OPD unit and other recently reported photometric based nitrite sensing platforms. It shows that our proof-of-concept detection system already has a promising limit of detection. Taking the step towards a fully integrated organic-semiconductor chip, our approach has the potential for parallel mass fabrication. Whereas the proof of concept was successfully performed, the analyte consumption was still rather high due to the large fluid chamber. The filling of the chamber requires approximately 100 µL to avoid light scattering due to air–liquid boundaries. Furthermore, operating the current setup in sunlight results in a significant photocurrent offset. This background signal can be subtracted in the post processing of the raw data or simply suppressed by using a non-transparent microfluidic device. Recently, we suggested employing a black absorptive material combined with a PDMS microfluidic for successful suppression of stray light [30]. Consequently, we aim to integrate the OLED–OPD matrix with a microfluidic device as the next step. Since the requirements for PON applications implicate a portable and wireless system, we also aim to upgrade the OLED–OPD sensing platform to a battery-powered system. A reliable hardware setup can be achieved by integrating low-cost, state-of-the-art hardware, i.e., a transimpedance amplifier for an I/V conversion and an analog-to-digital converter (ADC) that translates the photocurrent in a digital dataset representing the magnitude of the induced current. The results can be processed with a microcontroller and transmitted to a monitor. Wang et al. recently reported a comprehensive design of a hardware circuit for an optoelectronic sensor [41].

Further performance improvements of the system can be achieved by tuning the organic stacks of the OLED–OPD unit. Green OLEDs with higher efficiency have been recently proposed [42].

## 3. Conclusions

We developed the first photometric nitrite sensor based on a fully organic optoelectronic chip. The organic composition of the OLED–OPD matrix was designed to have suitable peak positions for high absorbance measurements of the azo dye (Abs. ~540 nm). We demonstrated a measurement of premixed nitrite standard solutions of different concentrations utilizing the Griess reagent. The calibration plot obtained from these measurements was found to be linear up to 1.2 mg/L, offering a linearity of 99%. The limit of detection (LOD) was calculated to be 46 µg/L (1.0 µM). This nitrite sensing unit is now ready for toxic threshold testing of drinking water or aquaculture systems. For quantification of concentrations below the current LOD, further improvements shall be investigated. As the next step, tuning of the organic stacks may serve for improvement of the system performance. The analyte consumption may be reduced by integrating the organic chip with a microfluidic unit. For Point-of-Need applications it is crucial to integrate the device with a portable and battery-powered system.

On the fabrication side, we already demonstrated the monolithic integration of 8 OLED–OPD pairs on a 12.5 mm by 12.5 mm substrate. Thus, this chip holds the potential for highly integrated multiplex sensing as well as the implementation of redundancy. In summary, employing organic optoelectronic devices in optical sensing approaches led to several benefits. The sensors can be manufactured on rigid or flexible substrates. The thermal evaporation technique enables a wide variety of geometrical shapes and device sizes leading to a high degree of design freedom. Particularly, the fabrication on flexible substrates is highly promising for large-scale roll-to-roll fabrication. The OLED–OPD unit holds great promise for use in a variety of applications ranging from bio-photonic sensors for human disease detection to environmental monitoring.

## Figures and Tables

**Table 1:** Sensing performance comparison of investigated OLED–OPD unit and recently reported photometric nitrite sensors.

Detection Hardware	LOD (µM)	Reference
Spectrometer based laboratory systems		
Fluorescence spectrometer	0.1	Zhang et al. [43]
Fluorescence spectrometer	0.04	Feng et al. [18]
Fluorescence spectrometer	0.05	Ren et al. [19]
UV–Vis spectrometer	0.1	Amanulla et al. [14]
UV–Vis spectrometer	0.12	Wang et al. [16]
Compact and miniaturized systems		
UV lamp–Photomultiplier tube	0.009	Wang and Wang [17]
Smartphone	0.65	Vidal et al. [15]
Raspberry Pi camera	4.34	Luka et al. [44]
Micro spectrometer	0.2	Greenway et al. [45]
LED–CCD camera	<250	Shi et al. [13]
LED–Photodiode	0.014	Sieben et al. [46]
LED–Photodiode	0.015	Beaton et al. [47]
LED–Photodiode	1.7	Khanfar et al. [20]
LED–Photodiode	1.54	Dudala et al. [21]
LED–Photodiode	1.7	Nightingale et al. [48]
LED–LED	0.74	Czugala et al. [23]
LED–PTB7:PC_70_BM OPD	<0.55	Pires et al. [49]
TCTA:Ir(mppy)_3_ OLED–DMQA:DCV3T OPD	1.0	This work
